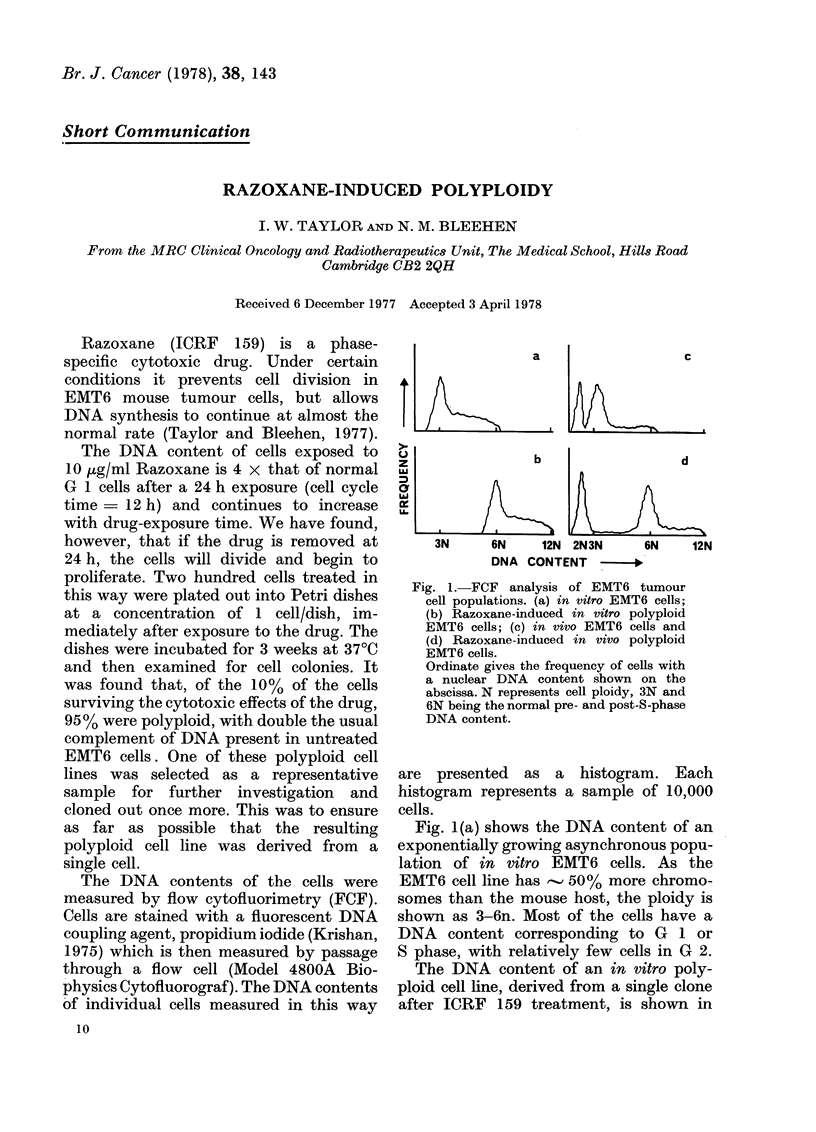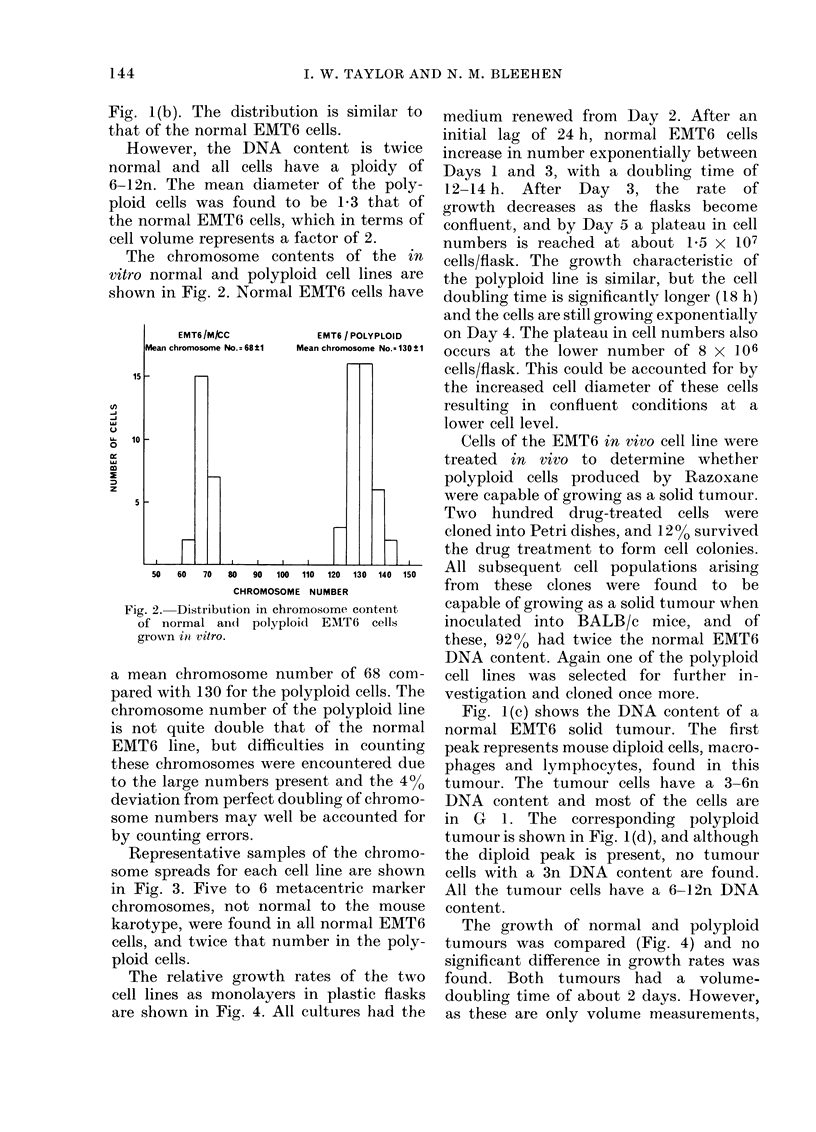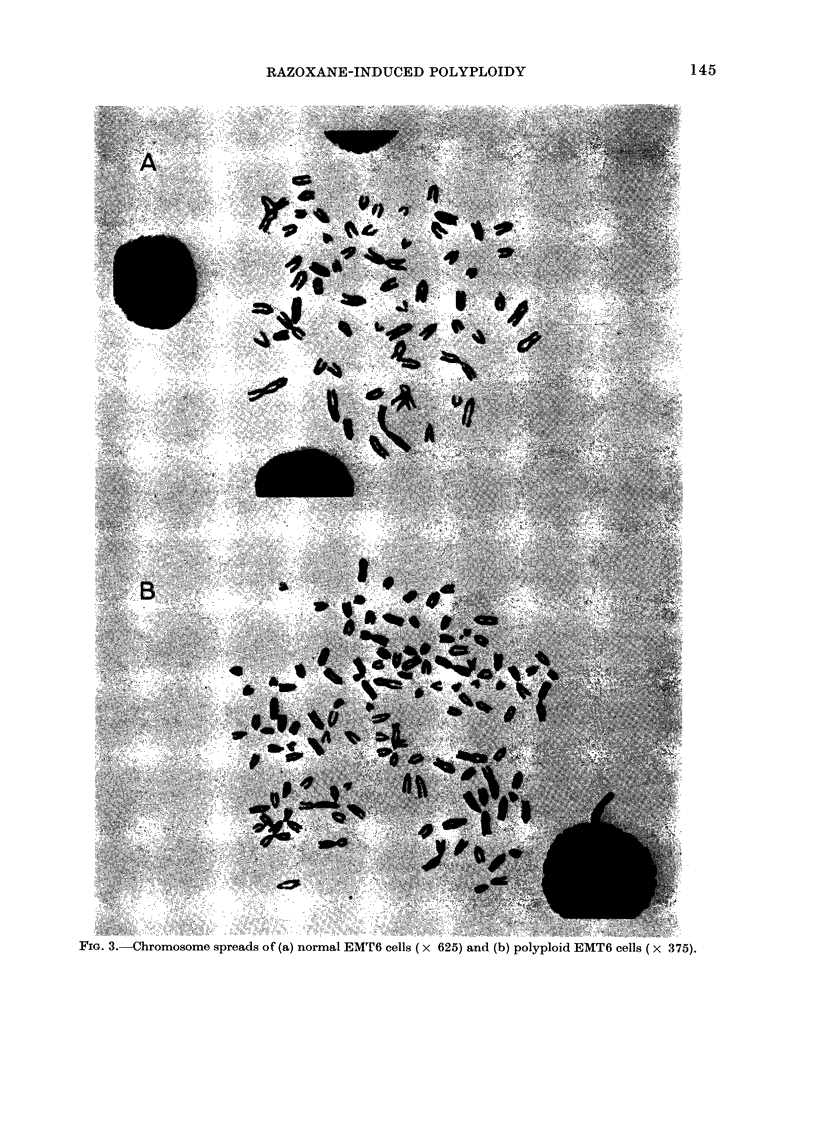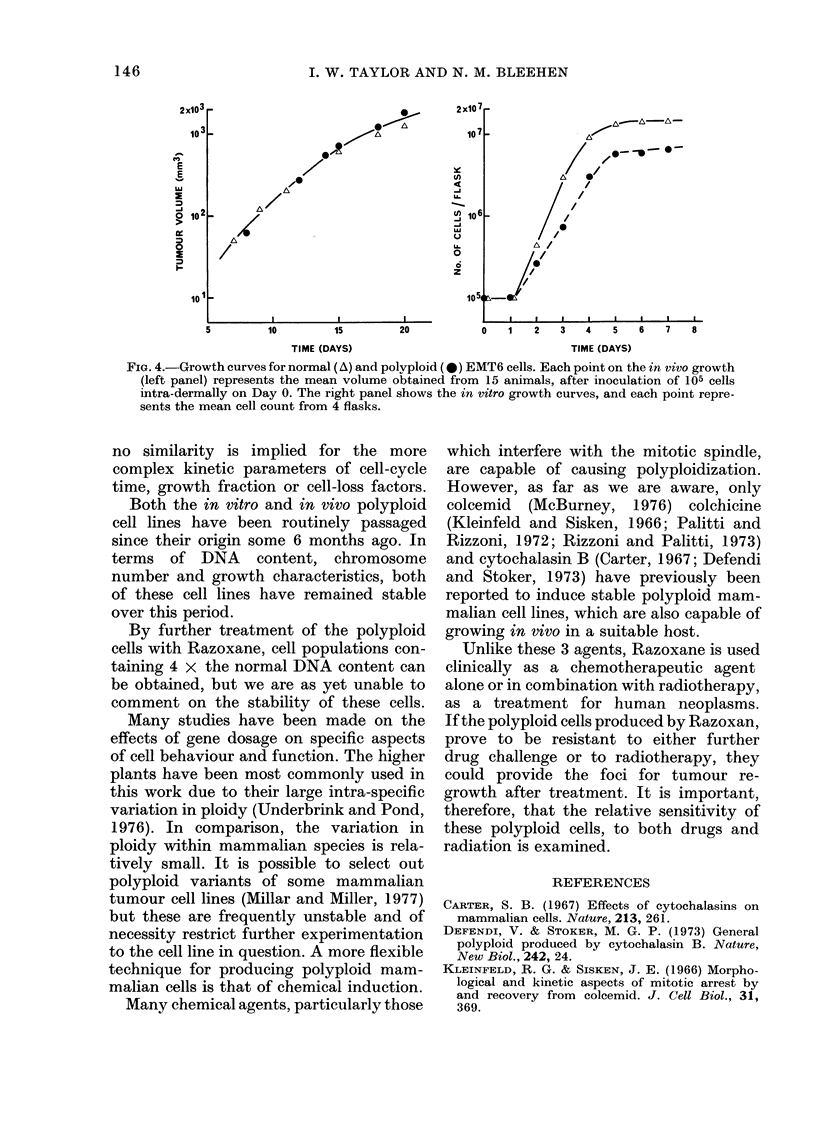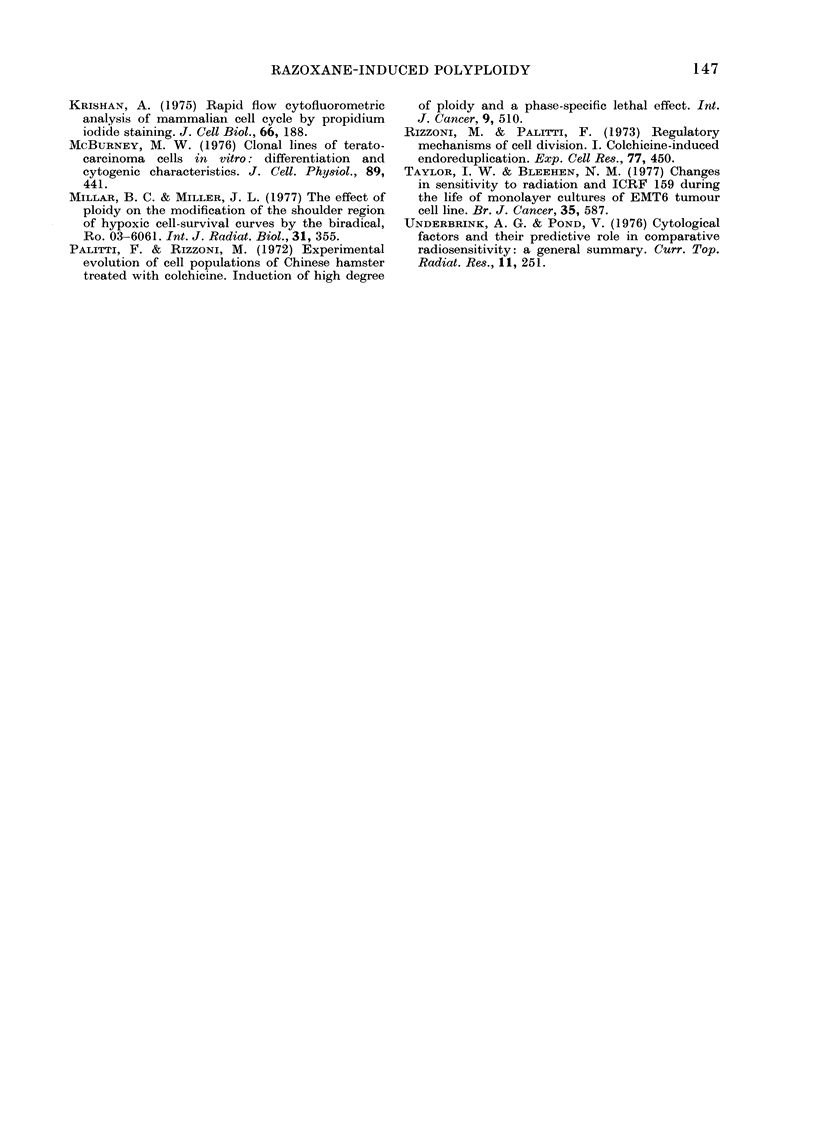# Razoxane-induced polyploidy.

**DOI:** 10.1038/bjc.1978.174

**Published:** 1978-07

**Authors:** I. W. Taylor, N. M. Bleehen

## Abstract

**Images:**


*Br. J. Cancer* (1978), **38**, 143

***Short Communication***

### RAZOXANE-INDUCED POLYPLOIDY

I. W. TAYLOR AND N. M. BLEEHEN

*From the MRC Clinical Oncology and Radiotherapeutics Unit, The Medical School, Hills Road Cambridge CB2 2QH*

Received 6 December 1977 Accepted 3 April 1978

Razoxane (ICRF 159) is a phase-specific cytotoxic drug. Under certain conditions it prevents cell division in EMT6 mouse tumour cells, but allows DNA synthesis to continue at almost the normal rate (Taylor and Bleehen, 1977).

The DNA content of cells exposed to 10 $\mu$g/ml Razoxane is 4 $\times$ that of normal G 1 cells after a 24 h exposure (cell cycle time = 12 h) and continues to increase with drug-exposure time. We have found, however, that if the drug is removed at 24 h, the cells will divide and begin to proliferate. Two hundred cells treated in this way were plated out into Petri dishes at a concentration of 1 cell/dish, immediately after exposure to the drug. The dishes were incubated for 3 weeks at 37°C and then examined for cell colonies. It was found that, of the 10% of the cells surviving the cytotoxic effects of the drug, 95% were polyploid, with double the usual complement of DNA present in untreated EMT6 cells. One of these polyploid cell lines was selected as a representative sample for further investigation and cloned out once more. This was to ensure as far as possible that the resulting polyploid cell line was derived from a single cell.

The DNA contents of the cells were measured by flow cytofluorimetry (FCF). Cells are stained with a fluorescent DNA coupling agent, propidium iodide (Krishan, 1975) which is then measured by passage through a flow cell (Model 4800A Biophysics Cytofluorograf). The DNA contents of individual cells measured in this way are presented as a histogram. Each histogram represents a sample of 10,000 cells.

Fig. 1.—FCF analysis of EMT6 tumour cell populations. (a) *in vitro* EMT6 cells; (b) Razoxane-induced *in vitro* polyploid EMT6 cells; (c) *in vivo* EMT6 cells and (d) Razoxane-induced *in vivo* polyploid EMT6 cells.
Ordinate gives the frequency of cells with a nuclear DNA content shown on the abscissa. N represents cell ploidy, 3N and 6N being the normal pre- and post-S-phase DNA content.

Fig. 1(a) shows the DNA content of an exponentially growing asynchronous population of *in vitro* EMT6 cells. As the EMT6 cell line has $\sim$ 50% more chromosomes than the mouse host, the ploidy is shown as 3–6n. Most of the cells have a DNA content corresponding to G 1 or S phase, with relatively few cells in G 2.

The DNA content of an *in vitro* polyploid cell line, derived from a single clone after ICRF 159 treatment, is shown in

Fig. 1(b). The distribution is similar to that of the normal EMT6 cells.

However, the DNA content is twice normal and all cells have a ploidy of 6–12n. The mean diameter of the polyploid cells was found to be 1·3 that of the normal EMT6 cells, which in terms of cell volume represents a factor of 2.

The chromosome contents of the *in vitro* normal and polyploid cell lines are shown in Fig. 2. Normal EMT6 cells have

Fig. 2.—Distribution in chromosome content of normal and polyploid EMT6 cells grown *in vitro*.

a mean chromosome number of 68 compared with 130 for the polyploid cells. The chromosome number of the polyploid line is not quite double that of the normal EMT6 line, but difficulties in counting these chromosomes were encountered due to the large numbers present and the 4% deviation from perfect doubling of chromosome numbers may well be accounted for by counting errors.

Representative samples of the chromosome spreads for each cell line are shown in Fig. 3. Five to 6 metacentric marker chromosomes, not normal to the mouse karotype, were found in all normal EMT6 cells, and twice that number in the polyploid cells.

The relative growth rates of the two cell lines as monolayers in plastic flasks are shown in Fig. 4. All cultures had the medium renewed from Day 2. After an initial lag of 24 h, normal EMT6 cells increase in number exponentially between Days 1 and 3, with a doubling time of 12–14 h. After Day 3, the rate of growth decreases as the flasks become confluent, and by Day 5 a plateau in cell numbers is reached at about $1{\cdot}5 \times 10^7$ cells/flask. The growth characteristic of the polyploid line is similar, but the cell doubling time is significantly longer (18 h) and the cells are still growing exponentially on Day 4. The plateau in cell numbers also occurs at the lower number of $8 \times 10^6$ cells/flask. This could be accounted for by the increased cell diameter of these cells resulting in confluent conditions at a lower cell level.

Cells of the EMT6 *in vivo* cell line were treated *in vivo* to determine whether polyploid cells produced by Razoxane were capable of growing as a solid tumour. Two hundred drug-treated cells were cloned into Petri dishes, and 12% survived the drug treatment to form cell colonies. All subsequent cell populations arising from these clones were found to be capable of growing as a solid tumour when inoculated into BALB/c mice, and of these, 92% had twice the normal EMT6 DNA content. Again one of the polyploid cell lines was selected for further investigation and cloned once more.

Fig. 1(c) shows the DNA content of a normal EMT6 solid tumour. The first peak represents mouse diploid cells, macrophages and lymphocytes, found in this tumour. The tumour cells have a 3–6n DNA content and most of the cells are in G 1. The corresponding polyploid tumour is shown in Fig. 1(d), and although the diploid peak is present, no tumour cells with a 3n DNA content are found. All the tumour cells have a 6–12n DNA content.

The growth of normal and polyploid tumours was compared (Fig. 4) and no significant difference in growth rates was found. Both tumours had a volume-doubling time of about 2 days. However, as these are only volume measurements,

FIG. 3.—Chromosome spreads of (a) normal EMT6 cells (× 625) and (b) polyploid EMT6 cells (× 375).

FIG. 4.—Growth curves for normal (Δ) and polyploid (●) EMT6 cells. Each point on the *in vivo* growth (left panel) represents the mean volume obtained from 15 animals, after inoculation of $10^5$ cells intra-dermally on Day 0. The right panel shows the *in vitro* growth curves, and each point represents the mean cell count from 4 flasks.

no similarity is implied for the more complex kinetic parameters of cell-cycle time, growth fraction or cell-loss factors.

Both the *in vitro* and *in vivo* polyploid cell lines have been routinely passaged since their origin some 6 months ago. In terms of DNA content, chromosome number and growth characteristics, both of these cell lines have remained stable over this period.

By further treatment of the polyploid cells with Razoxane, cell populations containing 4 × the normal DNA content can be obtained, but we are as yet unable to comment on the stability of these cells.

Many studies have been made on the effects of gene dosage on specific aspects of cell behaviour and function. The higher plants have been most commonly used in this work due to their large intra-specific variation in ploidy (Underbrink and Pond, 1976). In comparison, the variation in ploidy within mammalian species is relatively small. It is possible to select out polyploid variants of some mammalian tumour cell lines (Millar and Miller, 1977) but these are frequently unstable and of necessity restrict further experimentation to the cell line in question. A more flexible technique for producing polyploid mammalian cells is that of chemical induction.

Many chemical agents, particularly those which interfere with the mitotic spindle, are capable of causing polyploidization. However, as far as we are aware, only colcemid (McBurney, 1976) colchicine (Kleinfeld and Sisken, 1966; Palitti and Rizzoni, 1972; Rizzoni and Palitti, 1973) and cytochalasin B (Carter, 1967; Defendi and Stoker, 1973) have previously been reported to induce stable polyploid mammalian cell lines, which are also capable of growing *in vivo* in a suitable host.

Unlike these 3 agents, Razoxane is used clinically as a chemotherapeutic agent alone or in combination with radiotherapy, as a treatment for human neoplasms. If the polyploid cells produced by Razoxan, prove to be resistant to either further drug challenge or to radiotherapy, they could provide the foci for tumour regrowth after treatment. It is important, therefore, that the relative sensitivity of these polyploid cells, to both drugs and radiation is examined.

### REFERENCES

CARTER, S. B. (1967) Effects of cytochalasins on mammalian cells. *Nature*, **213**, 261.

DEFENDI, V. & STOKER, M. G. P. (1973) General polyploid produced by cytochalasin B. *Nature, New Biol.*, **242**, 24.

KLEINFELD, R. G. & SISKEN, J. E. (1966) Morphological and kinetic aspects of mitotic arrest by and recovery from colcemid. *J. Cell Biol.*, **31**, 369.

KRISHAN, A. (1975) Rapid flow cytofluorometric analysis of mammalian cell cycle by propidium iodide staining. *J. Cell Biol.*, **66**, 188.

MCBURNEY, M. W. (1976) Clonal lines of teratocarcinoma cells *in vitro*: differentiation and cytogenic characteristics. *J. Cell. Physiol.*, **89**, 441.

MILLAR, B. C. & MILLER, J. L. (1977) The effect of ploidy on the modification of the shoulder region of hypoxic cell-survival curves by the biradical, Ro. 03–6061. *Int. J. Radiat. Biol.*, **31**, 355.

PALITTI, F. & RIZZONI, M. (1972) Experimental evolution of cell populations of Chinese hamster treated with colchicine. Induction of high degree of ploidy and a phase-specific lethal effect. *Int. J. Cancer*, **9**, 510.

RIZZONI, M. & PALITTI, F. (1973) Regulatory mechanisms of cell division. I. Colchicine-induced endoreduplication. *Exp. Cell Res.*, **77**, 450.

TAYLOR, I. W. & BLEEHEN, N. M. (1977) Changes in sensitivity to radiation and ICRF 159 during the life of monolayer cultures of EMT6 tumour cell line. *Br. J. Cancer*, **35**, 587.

UNDERBRINK, A. G. & POND, V. (1976) Cytological factors and their predictive role in comparative radiosensitivity: a general summary. *Curr. Top. Radiat. Res.*, **11**, 251.